# Evaluation of plant and animal products against *Chilo partellus* Swinhoe (Lepidoptera: Crambidae) infestation in sorghum field

**DOI:** 10.1371/journal.pone.0319097

**Published:** 2025-04-24

**Authors:** Adem Nega, Emana Getu

**Affiliations:** 1 Department of Biology, College of Natural and Computational Sciences, Debre Markos University, Debre Markos,Ethiopia; 2 Department of Zoological Sciences, College of Natural and Computational Sciences, Addis Ababa University, Addis Ababa, Ethiopia; Gomal University, PAKISTAN

## Abstract

Plant and animal products with optimum concentrations were synthesized and evaluated against *Chilo partellus* Swinhoe infestation in the sorghum field. The experiment was designed in a randomized complete block design in a factorial arrangement with three replications. The treatments were *M. ferruginea* aqua extract, Cow urine, and a mixture of *M. ferruginea* and Cow urine each at three rates of concentration (5%, 10%, and 15%). Treatments were applied at two levels of frequencies (2 and 3 times application). The pest infestations and all its activities were systematically screened as well. Results indicated that all formulations significantly (P<0.01) reduced damage at the highest rates with three times applications. The mixture treatments were most effective in reducing leaf damage, dead hearts and the pest infestations with significant larval mortality at a higher rate with three times applications. No larvae per plant were recorded on sorghum treated with *M. ferruginea* aqua extract at a higher rate in three applications. Likewise, the highest mortality was recorded at the higher rate (15% concentration) of the mixture. Application rates and frequencies of all treatments were positively correlated to larval mortality. *M. ferruginea* aqua extract and the mixture were negatively correlated while cow urine has no relation to the density of larvae. Significantly taller plants, a smaller number of holes per plant, and the shortest tunnel length were recorded from the mixture, and *M. ferruginea* aqua extract treated plots at a higher rate and two times more applications. Best grain yields and yield advantages were recorded at three times applications of the higher rates. In conclusion, plant and animal-based insecticides at the higher rates with three times applications were best in reducing *C. partellus* infestations in the field. Likewise, their effect on the main natural enemy of the pest was minimal.

## Introduction

The exotic *Chilo partellus* Swinhoe is the most important pest of sorghum in Ethiopia. The insect infests sorghum crops throughout its growth stages in fields predominantly at lower altitudes and warm areas of the country [1]. Early instar larvae feed on young leaves in the whorl, which causes “a dead heart”, while mature larvae bore into the stems. In severe cases of infestations, plant growth is retarded; flowering and grain production are drastically reduced resulting in significant yield loss [2].

The application of chemical insecticides has been recommended to protect the sorghum crop from *C. partellus* attack [3,4]. However, chemicals are too expensive for subsistence farmers. They are also the cause of environmental and health hazards if not used judiciously or with proper safety measures [5,6]. Pesticides that have sub-lethal toxicity to target pests, but still kill natural enemies of the pests may cause target pests to increase, resulting in even higher yield losses [7,8]. So, due to these and other associated problems scientists have spurred the search for new strategies for managing the pest with minimal effects on natural enemies.

Bio-pesticides from plants and animals are desirable alternatives to synthetic insecticides for controlling pests. They are cheap, readily available, and affordable which can be an important option for resource-poor farmers of developing countries like Ethiopia. Bio-pesticides are best suited for use in organic food production and play a great role, especially in developing countries as a new class of eco-friendly products in controlling pests [9–11]. In most cases, their bioactive compounds are fairly complex groups, thereby making it more difficult for the pest to develop resistance [12–14]. Moreover, they are considered to be environmentally friendly and also reduce the cost of insecticides in pest management [6].

In Ethiopia, the use of these insecticides for field crops is not new, as it has been widely used by small-scale subsistence farmers for several years. However, their rate and frequency of application are not well investigated under field conditions. Thus, the objective of the present study was to determine the effect of rates and frequency of applications of the tested plant and animal-based insecticides on *C. partellus* infestations under field conditions.

## Materials and methods

### Study area

The present study was conducted during the main cropping season in 2017/2018 at Chorissa Kebele of Kombolcha town, in a farmer’s sorghum field. Kombolcha is located in Northeastern Ethiopia, South Wollo zone, 375 km from Addis Ababa. Its astronomical location is at 11°5′ N latitude and 39°44′ E longitude, with an elevation of 1842 meters above sea level. The site receives 1020 mm mean annual rainfall, but there are many variations in distribution and amount. The topography is lowland, which represents an arid and semi-arid ecology.

### Temperature, humidity and rainfall

The average annual minimum and maximum temperature ranges from 13.2–26.3 with a mean relative humidity of 73.3 (%). The site receives 1020mm mean annual rainfall but with many variations in distribution and amount. The topography is lowland type representing an arid and semi-arid ecology (Kombolcha Weather Stations).

### Botanicals collection and preparations

Seeds of *M. ferruginea* Hochst were collected from Addis Ababa University, Arat-kilo Campus, and roadside and grown voluntarily in plantations in Dessie town, Ethiopia. Seeds were washed, dried, and ground into fine powder manually using a homemade mortar and pestle [15]. The serial concentrations were prepared by mixing 50g, 100g, and 150g powder in 1 liter of water and filtered through a double muslin cloth [16]. The solution was then worked out to be at 5%, 10%, and 15% concentration weight by volume (w/v). Likewise, 50ml, 100ml, and 150ml of cow urine were added to 1L of water to get a similar concentration. The treatment combination was prepared by mixing with equal volumes in a 1:1volume-by-volume (v/v) ratio. Liquid soap at 0.1% was added as an emulsifier and all treatments were ready for spray [7,10,17]. All treatments were compared with untreated checks. Cow urine was diluted with water at a ratio of 1:3 (v/v) to reduce the risk of sorghum leaves burning.

### Method of treatment application

Treatments were sprayed three weeks after crop emergence to the whorl of sorghum leaves using a hand-operated sprayer (BackPack with a tank capacity of 4 gal and single nozzle). The treatments were applied two and three times at three rates of application: 5, 10, and 15% concentration (see Table in S1 Table for details). The total number of treatments was ten including a check. Untreated plots were used as a control. All applications were conducted in the late afternoon at 10:30 AM of the day to avoid wind disturbance.

### Field layout and management

The study was carried out on the farmer’s field of 16 x 35m plot size. The farmer was given all the required inputs as well as the produce at harvest after the necessary data were taken. Following the first rain in June, local sorghum variety (var. Gedalet) was planted immediately after the land preparation. About 2 seeds were planted per hole, but after germination, they were subsequently thinned to one plant per hole. The field was divided into 5 equal blocks. Each block was divided into 9 plots where 4 treatments were allotted at random. Each plot had a size of 2.5 x 3.5m or 8.75m^2^ with a spacing of 30cm and 75cm between plants and rows, respectively. The natural infestation of this field (sorghum) was expected to be high, due to the high occurrence of *C. partellus* in the area. A few volunteer plants from last year’s crop and other alternative hosts distributed over the whole field were left undisturbed to enhance *C. partellus* infestation. No fertilizer was applied and all the cultural practices were accomplished as per the local recommendation except insecticide application.

### Data collection

Both pre- and post-treatment applications data were recorded. Pre-treatment data were recorded one day before the first treatment application. Post-treatment data per plot were recorded after 2 and 3 times spraying. The number of leaves damaged and dead hearts was recorded as observed over time. Plots were visually rated for *C. partellus* leaf damage using 0–6 rating scale: 0=no leaf damage 1=0–10%, 2=11–20%, 3=21–30%, 4=31–40%, 5=41–50%, and 6=>51% based on the method of Monga and Dejen [18,19]. A rating of 6 reflected a highly damaged leaf, whereas a rating of 0 indicated zero damage. Density, dead larvae, plant height, borer holes count, and stem tunnelling were determined by randomly selecting 2 infested plants per plot before harvest.

Two plants from each plot were randomly collected at maturity, threshed, sun-dried for one week, and weighed to calculate the average weight of grains. The yield was recorded on kg/treatment basis and % grain yield advantage (PGYA) was calculated using the formula [19] as follows:


$$GYA\%=\frac{\left(Meangrainyieldofthetreatedplots-meangrainyieldofuntreated\right)}{Meangrainyieldofuntreatedplot}\times 100$$

$$Deadheart\left(\%\right)=\frac{numberdeadheartinaplot}{Totalplantsinaplot}\times 100$$

$$Lengthoftunneling\left(\%\right)=\frac{Lengthoftunneling\left(cm\right)}{Totallengthofstem\left(cm\right)}\times 100$$

### Data analysis

Data were analyzed using the Proc GLM procedure of SAS (SAS, 9.1) [20]. Count data were subjected to Square-Root transformation (X+0.5)^1/2^ as described by Gomez and Gomez [21]. The transformed data were then subjected to analysis of variance (ANOVA). Significant means were separated by Fisher’s Least Significance Difference (LSD) at a 5% error level.

## Results and discussion

### Pre-treatment application infestation of *C. partellus* on sorghum

Infestations were high and all the plots were non-significant before treatment. After obtaining of pre-treatment data, plants showing damage symptoms were removed from the plots, and treatments were applied. Then post-treatment data were collected.

### The effect of rates and application frequencies of *M. ferruginea* aqua extract, Cow urine, and the mixture treatments on dead heart and % leaf damage

Field results indicated that *C. partellus* infestation was significantly low with a higher rate and applications of frequencies. *M. ferruginea* seed aqua extract was found to be highly effective in reducing % leaf damage and dead hearts at higher rates with two applications and the mixture treatment with three applications (Tables 1 and 2).

**Table 1:** Mean (±SE) dead hearts and leaf damage from *M. ferruginea* extract, mixture, and Cow urine treatments.

Treatments	Rate (%)	Damage score	Leaf damage (%)	Dead hearts
*M. ferruginea* extract	5	2.91±0.09^d^	11=20	0.18±0.18(0.82)^c^
	10	2.72±0.10^de^	11-20	0±0.00(0.71)^c^
	15	1.93±0.22^g^	0-10	0±0.00(0.71)^c^
Cow urine	5	3.13±0.05^b^	21-30	0.82±0.47(1.06)^b^
	10	2.97±0.05^c^	11-20	0.53±0.26(0.99)^bc^
	15	3.02±0.05^b^	21-30	0.27±0.26(0.85)^bc^
*M. ferruginea* + Cow urine	5	2.96±0.05^c^	11-20	0.0±0.0(0.71)^c^
	10	2.41±0.06^ef^	11-20	0.27±0.26(0.85)^bc^
	15	1.85±0.15^g^	0-10	0.0±0.00(0.71)^c^
Untreated control	–	3.62±0.11^a^	21-30	1.94±0.27(1.87)^a^
	MSE	0.03		0.14
	LSD_.05_	0.28		0.55

Means in a column followed by the same letter is not significantly different from each other at 5% significance level, (LSD).

* numbers in parentheses are transformed values. MSE – mean square error.

**Table 2:** Mean (±SE) dead hearts and leaf damage (%) from application frequency of *M. ferruginea* extract, mixture, and Cow urine treatments.

Treatments	Frequencies of applications			
	Application times	Damage score	Leaf damage (%)	Dead heart
*M. ferruginea* aqua extract	2x	2.1±0.14^b^	11-20	0.0±0.0 (0.71)^c^
	3x	1.5±0.20^c^	0-10	0.0±0.0 (0.71)^c^
Cow urine	2x	2.2±0.12^b^	11-20	0.81±0.32 (1.22)^b^
	3x	2.1±0.22^b^	11-20	0.0±0.0 (0.71)^c^
*M. ferruginea* + Cow urine	2x	2.0±0.17^b^	11-20	0.0±0.0 (0.71)^c^
	3x	1.2±0.17^d^	0-10	0.0±0.0 (0.71)^c^
Untreated control	–	3.62±0.11^a^	21-30	1.94±0.27 (1.54)^a^
	MSE	0.12		0.08
	LSD	0.49		0.41

Means in a column followed by the same letter is not significantly different from each other at a 5% significance level, (LSD).

* numbers in parentheses are transformed values. MSE – mean square error.

The reduced leaf damage and dead hearts may be related to the deterrence, antifeedant, inhibition of hormone, and enzyme activity of the local insecticides. Consistent with this study, aqueous extract of neem seed kernel (*Aadrichata indica* A. Juss), nutmeg (*Monodora myristica* Gaertn), Dunal and physic nut (*Jatroph curcas* L.), and castor oil (*Ricinus communis* L.) treated plots reduced percent dead heart and white heads compared to the untreated check [22]. A similar result was also reported by Islam *et al.* [10]. Where neem extracts of, *A. indica* at 15ml/L concentration reduced 38.38% and 58.08% dead heart and white head respectively. In addition, neem was found to attract many predators [14].

It is obvious from the present study that *M. ferruginea* aqua extract in combination with cow urine could also be effective in reducing infestation. This is because together with the enzymatic action of cow urine and complex bioactivity of the botanical, the mixture treatment (synergy) increases efficacy and may have a significant fatal effect on the insect resulting in lower infestation of sorghum crop. This finding is consistent with that of Barapatre and Lingappa who documented similar results on the effectiveness of cow urine along with various botanicals against *Spodoptera litura* (Fab.) and *Helicoverpa armigera* (Hub.) in groundnut and chickpea, respectively [23].

### The effect of rate and frequency of *M. ferruginea* aqua extract, Cow urine, and the mixture treatments on larval density per plant and dead larvae

#### Effect of application rates on larvae/plant and dead larvae.

Significant differences were observed among the different rates of treatments in the proportion of mean larval density and dead larvae (Table 3). The highest larval density was obtained in the plot treated with cow urine at the lower rate of 5% concentration and this was not significantly different from others at the lower rates. The lowest was recorded on sorghum treated with *M. ferruginea* aqua extract at a higher rate of 15%. At the higher rate of 15% concentration, the highest mortality was recorded in the plot treated with the mixture. The lowest mortality was recorded on sorghum treated with cow urine at the lower rate of 5% concentration.

**Table 3:** Mean (±SE) larvae per plant and dead larvae from different rates of *M. ferruginea* extract, mixture, and Cow urine applications.

	Rates of applications			Frequencies of applications		
Treatments	Rate (%)	Live larvae/plant	Dead larvae	Application times	Live larvae/plant	Dead larvae
*M. ferruginea* aqua extract	5	2.0±0.57 (1.55)^b^	1.0±0.57 (1.17)^ef^	2x	0.6±0.53 (0.98)^c^	1.5±0.65 (1.34)^c^
	10	1.5±0.28(1.40)^c^	1.8±0.92 (1.43)^d^	3x	0±0.00(0.71)^e^	1.7±0.76 (1.46)^b^
	15	0.8±0.43 (1.11)^d^	2.3±0.72 (1.65)^b^			
Cow urine	5	2.1±0.43 (1.62)^b^	0.7±0.32 (1.05)^f^	2x	1.2±0.69 (1.23)^b^	1.0±0.40 (1.18)^d^
	10	1.8±0.16 (1.52)^c^	1.2±0.72 (1.22)^e^	3x	1.2±0.80 (1.32)^b^	1.1±0.53 (1.22)^d^
	15	1.5±0.28(1.40)^c^	1.5±0.86 (1.33)^de^			
*M. ferruginea* + Cow urine	5	2.0±0.0 (1.58) ^b^	1.3±0.87 (1.26)^de^	2x	0.7±0.56 (1.07)^c^	1.7±0.75 (1.44)^b^
	10	1.5±0.28(1.33)^c^	2.1±1.09 (1.52)^c^	3x	0.3±0.40 (0.88)^d^	1.8±0.60 (1.56)^a^
	15	1.3±0.87(1.26)^cd^	2.6±0.16 (1.77)^a^			
Untreated control	–	3.3±0.43 (1.95)^a^	0±0.0 (0.71)^g^	–	3.3±0.43 (1.95)^a^	0±0.0 (0.71)^e^
	MSE	0.10	0.20		0.12	0.16
	LSD_.05_	0.47	0.66		0.51	0.58

Means in a column followed by the same letter is not significantly different from each other at a 5% significance level, (LSD).

* numbers in parentheses are transformed values. MSE - mean square error.

These results showed that sorghum plots treated with the mixture exhibited a significantly (P<0.05) lower number of larvae as indicated by fewer larvae feeding on. The possible explanation for this may be the odor or volatiles from *M. ferruginea* that were high and in combination with cow urine, the efficacy of the mixture increased (Synergism). The result of the study is consistent with the report of Shrinivas and Balikai [24]. However, it can also be noted that larvae feeding at all the rates and application times of treated plants was considerably lower than that of the untreated (control). These results are in agreement with those reported that fresh and dried fruit extracts of Persian lilac at the rates of 2, 10, and 20 kg/ha and 1, 2, and 10 kg/ha were found to be effective in reducing the number of larvae per plant compared to the untreated (control) [25].

#### Effect of application frequencies on larvae per plant and dead larvae.

A higher number of larvae per plant were recorded at both applications in plots treated with cow urine (Table 3). The lowest mean number of live larvae was recorded in the plot treated with the mixture and no live larvae were recorded in plots treated with *M. ferruginea* aqua extract at the higher rate of three times applications. There was no significant difference (P<0.05) in the mean number of dead larvae in these treatments’ frequencies of applications. However, the highest number of dead larvae was found in the plot treated with the mixture at a frequency of three applications at a higher rate.

The reason for the better efficacy of the mixture treatment on *C. partellus* mortality could be treatment insecticides efficacy increased as the treatments were mixed and are more potent against this pest. In this study, the addition of a little soap to this treatment may stick the toxic and antifeedant activity effect of *M. ferruginea* seed aqua extract and cow urine to the leaf surface, preventing the insect from feeding and leading to death via starvation as well. This result agrees with those reported that a combination of *A. indica* + Karate 5 EC was effective against larvae of Fall armyworm (FAW), *Spodoptera frugiperda* (Smith) within the second round spraying [26].

#### Correlation relations among treatments rates, frequencies, larval density and mortality.

Application rates and frequencies of the treatments were positively related to larval mortality and they were not significant. Application rates of the treatments were negatively related to the density of larvae per plant. *M. ferruginea* aqua extract and *M. ferruginea* + Cow urine applications of frequencies were negatively related to the density of larvae while cow urine had no relationship and they were significant at 0.01 level.

The positive correlation recorded on the dead larvae at different treatment rates and frequencies indicated that mortality increases as the rate increases with increasing applications. A negative correlation indicated that the density of larvae decreases as the rate increases or vice versa. Zero correlation indicates that the density of larvae has no relationship with the rate and times of applications.

### The effect of rate and frequency of *M. ferruginea* aqua extract, Cow urine, and the mixture treatments on plant height, exit holes and tunnel length

#### Effect of application rates on plant height, exit holes and tunnel length.

There were significant differences (P<0.05) among different rates of applications in all treatments (Table 4). The tallest plants were recorded in plots treated with *M. ferruginea* seed aqua extract at the higher rate of 15% concentration. In contrast, the plots treated with cow urine resulted in shorter plants. Compared to the untreated, the treated plots had significantly taller plants.

**Table 4:** Mean (±SE) plant height, exit holes, and tunnel length from different rates of *M. ferruginea* extract, mixture, and Cow urine applications.

Treatments	Rate (%)	Plant height	Exit holes	Tunnel length
*M. ferruginea* aqua extract	5	252.3±0.32^b^	2.53±0.10 ^cd^	19.0±1.00^bc^
	10	250.0±0.57^c^	2.46±0.17^d^	18.6±0.87^c^
	15	253.6±1.20^a^	2.40±0.13^e^	17.0±1.52^d^
Cow urine	5	249.3±0.87 ^cd^	2.90±0.09^b^	22.0±0.57^b^
	10	247.0±1.15^d^	2.60±0.05^c^	21.3±0.87^b^
	15	251.0±0.57^c^	2.53±0.10 ^cd^	19.3±0.66^bc^
*M. ferruginea* + Cow urine	5	251.3±0.66^c^	2.47±0.06^d^	18.0±1.15^c^
	10	252.0±0.57^b^	2.59±0.16^c^	17.3±1.52^d^
	15	253.0±0.57^a^	2.33±0.12^f^	16.6±1.76^e^
Untreated control	–	213.0±5.76^e^	3.13±0.04^a^	23.7±0.87^a^
	MSE	29.08	0.03	4.15
	LSD_.05_	7.87	0.26	2.97

Means in a column followed by the same letter is not significantly different from each other at a 5% significance level, (LSD). MSE - mean square error.

A large number of exit holes per plant and the longest mean tunnel length were recorded in the plot treated with cow urine at the lower rate of 5% conc. and this was not statistically different from the control. However, the number declined at a higher rate of the mixture treatment.

Plant heights on treated plots were increased, and exit hole and tunnel lengths were decreased as compared with the untreated plots. However, the result indicated that in all the treatments height of plants at the higher concentrations sprayed were significantly taller than that of the lower rate and the untreated (control) implying that the persistent effect of this treatment may be produced from the higher dose and prevents the plant from insect damage. Likewise, the number of exit holes and length of the stem tunnel decreased as the dose increased. The current findings agree with the research results of Tilahun and Azerefegne who reported that among the *Milletia* treatments height of 5% and 3% sprayed on maize plants was significantly higher than that of 1% sprayed, but 1% of sprayed plants were significantly taller than those from the untreated plots [27].

#### Effect of application frequencies on plant height, exit holes, and tunnel length.

Plant height increases as the application rate increases in all treatments. Particularly, the tallest plants were recorded on sorghum treated with the mixture (Cow urine + *M. ferruginea* extract) at a higher rate with three applications. A significant (P<0.05) number of exit holes was recorded in the plot treated with cow urine at the lower rate of three applications while the lowest was the plot treated with *M. ferruginea* aqua extract with two applications at the higher rate (Table 5). Significant (P<0.05) longest tunnel length was recorded on sorghum treated with cow urine at the lower rates with two applications while the shortest was on sorghum treated with *M. ferruginea* aqua extract at the higher rates with two applications.

**Table 5:** Mean (±SE) plant height, exit holes, and tunnel length from application frequency of *M. ferruginea* extract, mixture, and Cow urine treatments.

Treatments	Frequencies of applications			
	Application times	Plant height	Exit holes	Tunnel length
*M. ferruginea* aqua extract	2x	254.0±1.00^b^	2.25±0.16^f^	15.6±1.04^f^
	3x	254.6±0.76^b^	2.33±0.10^e^	17.0±1.00^d^
Cow urine	2x	251.3±1.04^d^	2.53±0.09^c^	21.0±0.57^b^
	3x	253.0±0.49^c^	2.60±0.05^b^	19.0±1.00^c^
*M. ferruginea* + Cow urine	2x	254.3±0.76^b^	2.33±0.10^e^	16.0±1.00^e^
	3x	255.6±0.76^a^	2.40±0.05^d^	17.3±0.76^d^
Untreated control	–	213.0±5.76^e^	3.13±0.04^a^	23.7±0.87^a^
	MSE	2.34	0.78	1.43
	LSD.05	2.23	1.28	1.75

Means in a column followed by the same letter is not significantly different from each other at a 5% significance level, (LSD). MSE - mean square error.

Stem exit holes were reduced at higher rates with two applications in plots treated with the *M. ferruginea* aqua extract and the mixture treatments. This implies that two applications of these treatments at a higher rate were sufficient to provide complete protection of sorghum from the larvae attack. The possible explanation could be natural insecticides with repeated applications at higher doses may prevent the insect from further feeding or attacking. In agreement with this result, the reduction of insect damage to crops with more frequent insecticide applications was reported in many previous studies [28,29]. Similarly, Neem seed powder at the rate of 0.65–1gm with frequencies of 3–4 times applications provided substantial damage reduction and yield increments of sorghum [19]. However, there was no significant difference in larval tunnel length among the different rates and frequencies of all the treatments and the untreated plots. This might be because the late instar larvae that hide in sorghum stem and are protected from the treatment effect made tunnels.

### The effect of rates and application frequencies of *M. ferruginea* aqua extract, Cow urine, and the mixture treatments on grain yield and yield advantage

Grain yields and yield advantages vary among the different treatment rates and frequencies of applications (Table 6). The highest yield and yield advantage were recorded in the plot of the mixture at the higher rate of 3 times applications. But this was not statistically different from *M. ferruginea* aqua extract. In treated plots, the entire rates gave high grain yield advantage as the rate increased.

**Table 6:** Mean (±SE) yield (kg/t) and yield advantage (%) from different rates and application frequency of *M. ferruginea* extract, mixture, and Cow urine treatments.

Rates of applications				Frequencies of applications		
Treatments	Rate (%)	Yield kg/t	Yield advantage (%)	Application times	Yield kg/t	Yield advantage (%)
*M. ferruginea* aqua extract	5	1.82±0.21^e^	50.4	2x	2.29±0.21^d^	89.2
	10	1.94±0.27^d^	60.3	3x	2.31±0.20^c^	90.9
	15	2.28±0.2^b^	88.4			
Cow urine	5	1.76±0.15^ef^	45.5	2x	2.0±0.24^e^	65.3
	10	1.98±0.15^c^	63.6	3x	1.96±0.30^ef^	61.9
	15	1.94±0.24^d^	60.3			
*M. ferruginea* + Cow urine	5	1.85±0.18^e^	52.8	2x	2.32±0.06^b^	91.7
	10	1.95±0.12 ^cd^	61.1	3x	2.33±0.02^a^	92.5
	15	2.30±0.15^a^	90.0			
Untreated control	–	1.21±0.13^g^	–	–	1.21±0.13^g^	–
	MSE	0.14		MSE	0.14	
	LSD	0.55		LSD	0.55	

Means in a column followed by the same letter are not significantly different at 5% significance level, (LSD). MSE - mean square error.

The results from the present study showed that plant and animal-based insecticides increase sorghum yield by lowering the population of *C. partellus* in the field. The highest yield of sorghum was obtained in plots treated with the mixture and *M. ferruginea* seed aqua extract at the higher rates of three times applications. The good performance with respect to yield by these treatments was due to the suppression of the pest population which has contributed to the increase in sorghum yield. Similar to this result physic nut seed powder, pyrethrum flower powder, tobacco leaf powder, neem seed powder, and *E. schmperiana* leaf powder at the rate of 0.65-1.0g and frequency of 3–4 times application resulted in yield increment of 46–69%, 48–55%, 51–56%, 31–53%, and 39–57% of sorghum over the untreated (control), respectively [19]. Similarly, a higher yield was protected from *B. fusca* attack by *M. ferruginea* treatment. The highest (97%) yield was obtained from plots treated with *M. ferruginea* at 5% concentration [28].

## Conclusion

This study clearly indicated that all insecticides tested were effective in reducing *C. partellus* infestations on sorghum crops under field conditions. Likewise, the aqua extract of *M. ferruginea* and the mixture were effective in reducing leaf damage, dead hearts, and larvae abundance per plant. These treatments caused significant larval mortality at higher rates with three times applications. The present study confirms that *C. partellus* infestations and all its activities can be protected in the field using these bio-pesticides. Hence, it can be concluded that plant and animal-based insecticides applied at higher rates with a frequency of two and three applications were best in controlling the pest infestations in the field. Above all, the effect of these insecticides on the natural enemy of *C. partellus* was minimal. Therefore, it was recommended that plant animal-based insecticides be used as a management option for *C. partellus* as components of integrated pest management.

## Supporting information

S1 Table Treatment details for *Chilo partellus* management: treatments, rates, application interval, and application times. (DOCX)

S2 Table Relationship between treatments, larvae per plant, rates, and frequency. (DOC)

## References

[1] Getu E, Overholt W, Kairu E, Macopiyo L, Zhou G. Predicting the distribution of *Chilo partellu*s (Swinhoe) (Lepidoptera: Crambidae) and *Cotesia flavipes* (Cameron) (hymenoptera: Braconidae) in Ethiopia using correlation, step-wise regression, and geographic information system. Ins Sci Appl. 2002;22:123–9.

[2] FAO, Food and Agriculture Organization. Sorghum productivity practices in Sub-Saharan Africa. 2010.

[3] Gupta S, Handore K, Pandey I. Effect of insecticides against *Chilo partellus* (Swinhoe) damaging Zea mays (maize). International Journal of Parasitology Research. 2010;2(2):4.

[4] Ateia AMA. Efficacy evaluation of carbofuran, profenofos and fipronil insecticides against stem borer *Chilo partellus* (Swinhoe) on maize. Doctoral Dissertation, University of Gezira, 2018; 83 pp.

[5] Williamson S. The dependency syndrome: pesticide use by African smallholders: a report for PAN UK’s pesticides poverty and livelihoods project. Pesticide Action Network. 2003:96.

[6] Vitale J, Boyer T, Uaiene R, Sanders J. The economic impacts of introducing Bt technology in smallholder cotton production systems of West Africa: A case study from Mali. 2007.

[7] Thippeswamy C, Venkatesh H, Shivasharanappa Y. Evaluation of new insecticide molecules, botanicals and biopesticides against maize stemborer, *Chilo partellus* (Swinhoe) (Lepidoptera: Crambidae). International Journal of Plant Protection. 2010;3:124–6.

[8] Kipkoech AK, Schulthess F, Yabann WK, Kipsat MJ, Mithofer D. Measuring the economic value of redistributing parasitoids for the control of the maize stemborer *Busseola fusca* Fuller (Lepidoptera: Noctuidae) in Kenya. African Journal of Agricultural Research and Economics. 2010;4(2):140–58.

[9] Akob C, Ewete F. The efficacy of ashes of four locally used plant materials against *Sitophilus zeamais* (Coleoptera: Curculionidae) in Cameroon. International Journal of Tropical Insect Science. 2007;27(1):21–6.

[10] Islam M, Das S, Islam K, Rahman A, Huda M, Dash P. Evaluation of different insecticides and botanical extracts against yellow stem borer, *Scirpophaga incer* Tulas in rice field. International Journal of Biosciences. 2013;10:117–25.

[11] Kareru P, Rotich ZK, Maina EW. Use of botanicals and safer insecticides designed in controlling insects: the African case. Insecticides–Development of Safer and More Effective Technologies. 2013 Jan 30;10:297-309.

[12] FAO, Food and Agriculture Organization. Food and agricultural organization of the united nation. 2014. Available from: http://faostat.fao.org/site/291/default.aspx

[13] Khan I, Nawaz M, Said F, Subhanullah K. Integrated pest management of maize stem borer, *Chilo partellus* (Swinhoe) in maize crop and its impact on yield. Journal of Entomology and Zoology Studies. 2015;3(5):470–2.

[14] Wahedi J, David D, Danba E, Yisa S, Zakariya R. Yield performance of maize treated with neem seed extracts against stem borers. J Exp Agri Int. 2016;12(6):1–8.

[15] Jembere B. Evaluation of the toxicity potential of *Militia* *ferruginea* (Hochst) Baker against *Sitophilus zeamais* (Motsch.). International Journal of Pest Management. 2002;48:29–32.

[16] Venkat R, Devi S, Vishnu R, Reddy D. Evaluation of botanical and other extracts against plant hoppers in rice. Journal of Biopesticides. 2012;5:17–25.

[17] Berger A, Endod plant, Phytolacca dodecandra and its application in Bllharziosis control program, Klwe, Zambia. Natural Plants Products as Pesticides. Proceedings of the First National Symposium in Zambia, Lutsaka, Zambia. 1995;53–65.

[18] Moonga M. Assessment of infestation levels of *Chilo partellus* Swinhoe (Lepidoptera: Crambidae) on maize and the impact of its parasitoid, *Cotesia flavipes* Cameron (Hymenoptera: Braconidae) in Sinazongwe districts of Zambia [dissertation]. University of Zambia, Lusaka, 2007:57.

[19] Dejen A. On-farm bio-efficacy of *Azadirachta indica* and *Melia azedarach* seed powders on lepidpteran stem borers on sorghum in Northeastern Ethiopia. Biopesti. Int. 2008; 4: 57-62.

[20] SAS, Statistical Analysis System Software; Ver. 9.1. SAS Institute Inc., Carry. 2009.

[21] Gomez K, Gomez A. Statistical procedures for agricultural research. 2nd ed. John Wiley & Sons; 1984. p. 680.

[22] Amaugo GO, Emosairue SO. The efficacy of some indigenous medicinal plant extracts for the control of upland rice stem borers in Nigeria. Tropical and sub-tropical Agro-ecosystems. 2003;2(3).

[23] Barapatre A, Lingappa S. Larvicidal and antifeedant activity of indigenous plant protection practices for *Helicoverpa armigera* (Hub.). Proceedings of the National Symposium Frontier Areas of Entomological Research, New Delhi. 2003:1–53.

[24] Shrinivas M, Balikai R. Evaluation of plant products in combination with cow urine and panchagavya against sorghum shoot fly, *Atherigona** **soccata* Rondani. Karnataka Journal of Agricultural Sciences. 2009;3:618–20.

[25] Gebre-Amlak A, Azerefegne F. Insecticidal activity of Chinaberry, Endod and Pepper tree against the maize stalk borer, *Busseola fusca* Fuller (Lepidoptera: Noctuidae) in southern Ethiopia. Int J Pest Managt. 1999;1(1):9–13.

[26] Sisay B, Tefera T, Wakgari M, Ayalew G, Mendesil E. The Efficacy of Selected Synthetic Insecticides and Botanicals against Fall Armyworm, *Spodoptera frugiperda*, in Maize. Insects. 2019;10(2):45. doi: 10.3390/insects10020045 30717302 PMC6410260

[27] Tilahun B, Azerefegne F. Efficacy of the aqueous crude seed extract of *Millettia ferruginea* (Fabaceae) on the maize stemborer *Busseola fusca* (Lepidoptera: Noctuidae) in the field. Int J Trop Insect Sci. 2013;33(04):256–63. doi: 10.1017/s1742758413000258

[28] Story RN, Sundstrom FJ. Influence of cabbage cultivar and frequency of insecticide application on damage by the Cabbage Looper (Lepidoptera: Noctuidae). The Florida Entomologist. 1986;69(1):174. doi: 10.2307/3494759

[29] Ajeigbe H, Singh B. Integrated pest management in cowpea: effect of time and frequency of insecticide application on productivity. Crop Protection. 2006;25(9):920–5.

