# Supplementary material for: Evaluation of plant and animal products against Chilo partellus Swinhoe (Lepidoptera: Crambidae) infestation in sorghum field
Source: PLoS One. 2025 Apr 24;20(4):e0319097. doi: 10.1371/journal.pone.0319097 (PMC12021290; doi:10.1371/journal.pone.0319097)
Supplement: S1 Table — (DOCX) [file pone.0319097.s001.docx]

**Supporting information**

**S1 Table. Treatment details for *Chilo partellus* management: treatments, rates, application interval, and application times.**

| Treatments | Rates | Application interval | Applications times |
| --- | --- | --- | --- |
| *Milletiaferruginea*(Hochst)seed aqueous extracts | 5 %  10 %  15 % | Each per plot sprayed in 7 days interval | 2 and 3 times applied |
| Fermented Cow urine | 5 %  10 %  15 % | Each per plot sprayed in 7 days  interval | 2 and 3 times applied |
| Cow urine + *Milletiaferruginea*extract | - | The mixture in the ratio 1:1 was sprayed per plot in 7 days interval | 2 and 3 times applied |
| Sorghum (Untreated check) | - | - | - |

% = per cent
