# Supplementary material for: Evaluation of plant and animal products against Chilo partellus Swinhoe (Lepidoptera: Crambidae) infestation in sorghum field
Source: PLoS One. 2025 Apr 24;20(4):e0319097. doi: 10.1371/journal.pone.0319097 (PMC12021290; doi:10.1371/journal.pone.0319097)
Supplement: S2 Table — (DOC) [file pone.0319097.s002.doc]

**Supporting information**

**S2 Table. Relationship between treatments, larvae per plant, rates,** and frequency.

| Treatments |  | Larvae per plant | Rates | Frequency |
| --- | --- | --- | --- | --- |
| *M. ferruginea* aqua extract | Dead larvae | - | 0.363 (0.335) | 0.233 (0.176) |
|  | Rate | -0.529 (0.143) | - | - |
|  | Frequency | -0.333 (0.176) | - | - |
| Cow urine | Dead larvae | - | 0.333 (0.382) | 0.132 (0.600) |
|  | Rate | -0.548 (0.127) | - | - |
|  | Frequency | 0.000 (1.00) | - | - |
| *M. ferruginea* + Cow Urine | Dead larvae | - | 0.414 (0.268) | 0.218 (0.384) |
|  | Rate | -0.087 (0.824) | - | - |
|  | Frequency | -0.243 (0.332) | - | - |

Correlation is significant at 0.01 and 0.05 levels
